# A Practical Treatise on the Causes, Symptoms and Treatment of Spermatorrhœa

**Published:** 1848-04

**Authors:** 


ART. VII.—*A Practical Treatise on the Causes, Symptoms and Treatment of Spermatorrhœa.* By M. LALLEMAND, formerly Professor of Clinical Surgery at the University of Montpellier; Member of the Royal Academy of Medicine of Paris, etc. Translated and Edited by HENRY J. McDOUGALL, Member of the Royal College of Surgeons of England; Fellow of the Royal Medical and Chirurgical Society; and formerly House Surgeon to the University College Hospital. Philadelphia: Lea and Blanchard: 1848. 8vo. pp. 320.

This is an elaborate treatise on a subject but little studied, we incline to believe, by most medical men, and yet it is one which is worthy of serious attention. The author in his preface states, that during a period of fourteen years, he "has collected more than one hundred and fifty cases in which involuntary seminal discharges were sufficiently serious to disorder the health of the patients considerably, and even sometimes to cause death." Affections originating from this cause are so obscure as often to be mistaken. Generally they are referred to the brain or the stomach, and the remedies being addressed to those organs prove unavailing or mischievous.

These discharges present themselves under various conditions, differing much in their respective degrees of importance. Occurring spontaneously during sleep in a healthy and continent person, they are at least harmless. But they may become excessive, and so give rise to serious inconvenience, or, being excited by inordinate indulgence, may induce a state of irritation causing a hurried discharge of the secreted fluid, without complete erection, and almost without sensation. Lastly, it may go so far as to allow the expulsion of the semen without either erection or enjoyment, especially during defecation or the expulsion of the urine.

It is far from being true, as is generally believed, that diurnal pollutions are always the result of venereal excesses, or of vicious habits. This the author proves by many conclusive examples, and he shows that by a skilful diagnosis and a timely application of preventive or remedial measures many of these very distressing cases are easily cured. Very often they proceed from chronic catarrh or irritation of the bladder. Blennorrhagia is a frequent cause. Our author's remedy is given in the following extract:

"Not long since cauterization of the prostatic portion of the urethra was looked on as the extreme of rashness, so much was the introduction of the least particle of the nitrate of silver into the bladder dreaded; although these fears were only founded on argument, they were generally received, and seemed so natural that I was influenced by them for several years. I have stated in another place the means by which I shook off these foolish fears, and the successful results that have followed the application of the nitrate of silver to the mucous membrane of the bladder in catarrhal affections of that organ. Since that time, whenever I meet with cases in which the affection of the prostatic mucous membrane extends to that of the bladder, I begin by cauterizing the latter, and I continue the application as far as the bulb of the urethra while withdrawing the instrument, by inclining it rapidly to the right and left. It is not to take the length of the canal that I introduce a catheter in these cases, but in order to empty the bladder completely, so that the nitrate of silver may act with more energy. We have just seen the effect of this treatment: a patient who previously passed urine two or three times in the hour was enabled to retain the excretion as long as is usual, and at the same time the spermatorrhœa from which he suffered was cured."

Patients troubled with this affection micturate more frequently than natural. The kidneys are in a state of excitement, as are also the testicles. On the efficacy of the nitrate of silver in this excited state of the urinary apparatus, our author has the following remarks:

"It is by no means astonishing that in this state the application of the nitrate of silver to the prostatic mucous membrane should produce effects more direct and powerful than those of any other remedy. We know well how promptly and effectually nitrate of silver acts on tissues which are granular, injected or swollen from the effects of prolonged inflammation. Its results are especially evident in the chronic ophthalmia of scrofulous patients. Soon after the nitrate has been applied, the tissues empty themselves, contract, and become paler, and they retain an energetic action which preserves them from a relapse, to which the patients are often liable when a cure has been obtained by other means. On this account I have employed nitrate of silver in the chronic inflammation of the vagina and neck of the uterus, which keeps up leucorrhœal discharge in so many cases, and in chronic catarrh of the bladder, which is so difficult of cure by other means; and I have always had cause to be pleased with its action in these affections."

Other causes of spermatorrhœa are enumerated by the author, among which cutaneous affections appear to hold a prominent place, acting, however, rather as adjuvants than as the chief agent, for the causes of this disorder, according to Mr. L., rarely act singly. Diseases of the rectum may be mentioned as further causes. These are sometimes mechanical, as stricture; at other times, it is irritation of the bowel, from long continued diarrhea, or from hemorrhoids, constipation or ascarides. The following case, in which the latter cause kept up the discharges, is instructive:

"I have recently been consulted by one of my former pupils for a case in which the discharges were very serious, and had resisted the most various modes of treatment. They were attributed to masturbation, and the patient's confessions justified this opinion; yet a passage in his letter convinced me that a mistake had arisen on at least one point. After speaking of supposed hemorrhoids which irritated the margin of the anus, the patient added that the pain and itching he felt there was such that he often introduced his finger forcibly into the rectum, and had several times brought down ascarides on withdrawing it. This circumstance, previously neglected, caused me to think that the ascarides, if they had not caused the pollutions, at all events kept them up, and I prescribed accordingly, with success. We must remember, then, that the emissions may be kept up in persons who have practiced masturbation, by the presence of ascarides, even in cases in which these entozoa have not excited the habit; and on this account it is necessary to consider their presence with much attention."

But by far the most fruitful causes of this affection are

"abuse" and venereal excesses, melancholy and most disgusting examples of which are related by the author. We have not room to quote any of them.

The treatment must vary with the cause, and hence the importance of examining carefully into every case. When ascarides, structure of the rectum, hemorrhoids, constipation, or any such trouble is at the bottom of the disorder, the remedy is very simple. Oftener, however, it has its origin in chronic inflammation of the urethra, for which the other treatment is demanded. The remedy in such cases is cauterization, concerning which our author remarks as follows:

"This operation is especially indicated in cases of chronic inflammation, or irritation of the urethra; its results may be considered certain when involuntary discharges follow a common clap, or non-contagious gleet. I have also found it successful in many cases where atony, or relaxation seemed to predominate, and in a few cases of a marked nervous disorder, and genital pre-disposition. In the latter cases, however, the benefit derived from cauterization has seldom proved permanent, though I believe that by changing the condition of the tissues, the foundation has been laid for the successful use of other means.

Before proceeding to cauterization it is indispensably necessary to introduce a catheter; for the double purpose of taking the exact length of the urethra, and of completely emptying the bladder. On slowly withdrawing the instrument, during the escape of urine, the stream is arrested as soon as the eyes of the catheter enter the canal, and recommences when they are again pushed into the bladder. The penis being then moderately stretched, the thumb and fore finger should be applied to the instrument at the point of the glans. When the catheter is withdrawn, the distance between the finger and thumb and its eyes, gives the exact length of the urethra, and this must be immediately marked on the *porte-caustique*, the eyes of the catheter being applied to its olivary extremity, and the position of the fingers indicated by fixing a little slider on the stem of the instrument. When the *porte-castique* has penetrated so far into the urethra, that this slider touches a point of the glans—the penis being in exactly the same state of elongation in which it was when the catheter was introduced—it is clear that its olivary extremity will be in precisely the spot previously occupied by the eyes of the catheter, when the length of the canal was taken; that is to say, at the commencement of the neck of the bladder—a position which it is highly important to the operator to be assured of.

The bladder must be completely emptied, in order that no urine may penetrate into the tube of the *porte-caustique*, and that none may enter the urethra during cauterization. When the caustic is wetted by the urine, it acts much less energetically than if it were dry, and its action extends to parts where it was not required. The inflammation set up may, under these circumstances, be insufficient to fulfil the desired object, although at the same time, extremely painful on account of its extent."

The cauterization is practised to bring on a lasting change in the condition of the tissues, and ought not to be carried to the point of producing a slough. The author observes:

"It is now twenty years since I first commenced the practice of cauterizing the prostate in cases of ancient gleet, which had resisted all other kinds of treatment; very soon afterwards I applied the same powerful means to the treatment of involuntary seminal discharges. Since that time, I have performed the operation almost daily, and I have never seen, in my own practice, any of the violent effects, such as retention of urine, hemorhage, long-continued violent pain, narrowing of the passage, etc., described by some operators as supervening. Indeed, I should almost doubt the accuracy of these descriptions, had I not been consulted in one or two cases in which the symptoms had been set up. Such ill effects result from the injurious and absurd practice of some surgeons, of cauterizing the urethra during a fixed period of time, watch in hand. The time required to glance at the second hand, is more than sufficient for applying the caustic.

During the first days subsequent to cauterization, baths, enemata, and diluents should be prescribed, with milk and vegetable diet, so as to dilute the urine as much as possible. All fatigue should be abstained from, and exposure to cold rigorously avoided. For two or three days micturition is frequent, painful and accompanied with the escape of a few drops of blood. But these symptoms soon pass off, provided no imprudence be committed. I have, however, known the pain continue for ten days or more, in patients who committed errors of diet, or fatigued themselves too early, or who exposed themselves to cold or damp."

The patient and physician ought to be on their guard against expecting the curative effects to follow too soon. They can hardly be looked for earlier than a fortnight, and a month is necessary to decide whether the remedy has been effectual. The author records his opinion that but for the application of the nitrate of silver, in this manner, two thirds of the cases of spermatorrhœa would be incurable.

